# Molecular Imaging with ^18^F-FDG PET/CT and ^99m^Tc-MIBI SPECT/CT in Osteitis Fibrosa Cystica Generalisata

**DOI:** 10.3390/diagnostics11081355

**Published:** 2021-07-28

**Authors:** Adrien Holzgreve, Matthias P. Fabritius, Thomas Knösel, Lena M. Mittlmeier, Johannes Rübenthaler, Reinhold Tiling, Christoph J. Auernhammer, Peter Bartenstein, Marcus Unterrainer

**Affiliations:** 1 Department of Nuclear Medicine, University Hospital, LMU Munich, 81377 Munich, Germany; Lena.Mittlmeier@med.uni-muenchen.de (L.M.M.); Reinhold.Tiling@med.uni-muenchen.de (R.T.); Peter.Bartenstein@med.uni-muenchen.de (P.B.); 2 Department of Radiology, University Hospital, LMU Munich, 81377 Munich, Germany; Matthias.Fabritius@med.uni-muenchen.de (M.P.F.); Johannes.Ruebenthaler@med.uni-muenchen.de (J.R.); Marcus.Unterrainer@med.uni-muenchen.de (M.U.); 3 Institute of Pathology, Faculty of Medicine, LMU Munich, 81377 Munich, Germany; Thomas.Knoesel@med.uni-muenchen.de; 4 Department of Medicine IV, University Hospital, LMU Munich, 81377 Munich, Germany; Christoph.Auernhammer@med.uni-muenchen.de

**Keywords:** ^18^F-FDG PET/CT, ^99m^Tc-MIBI SPECT/CT, pitfall, osteitis fibrosa cystica generalisata, brown tumor, parathyroid adenoma

## Abstract

Benign so-called “brown tumors” secondary to hyperparathyroidism are a rare diagnostic pitfall due to their impressively malignant-like character in various imaging modalities. We present the case of a 65-year-old male patient with multiple unclear osteolytic lesions on prior imaging suspicious for metastatic malignant disease. Eventually, findings of ^18^F-FDG PET/CT staging and ^99m^Tc-MIBI scintigraphy resulted in revision of the initially suspected malignant diagnosis. This case illustrates how molecular imaging findings non-invasively corroborate the correct diagnosis of osteitis fibrosa cystica generalisata with the formation of multiple benign brown tumors.

**Figure 1:**
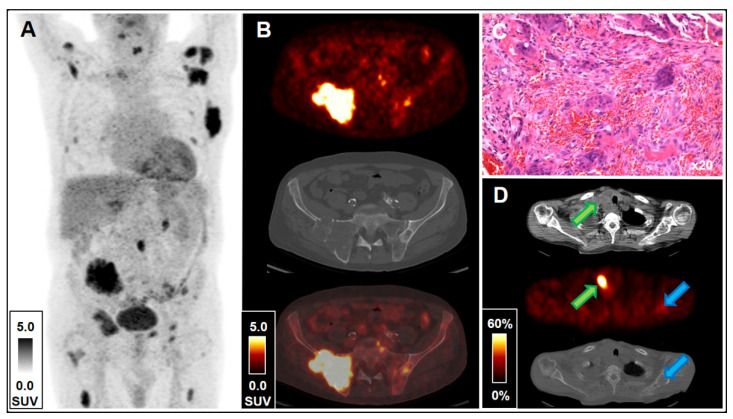
^18^F-FDG PET (**A**), ^18^F-FDG PET/CT (**B**), H&E staining (**C**), ^99m^Tc-MIBI SPECT/CT (**D**). A 65-year-old male patient with multiple unclear osteolytic lesions on CT imaging and suspected metastatic malignant disease of unknown primary presented for ^18^F-FDG PET/CT staging prior to biopsy. The patient history included chronic kidney disease (CKD) and pronounced secondary renal hyperparathyroidism on serum biochemistry, as well as COVID-19 one month earlier. Prophylactic shunt placement had previously been performed (Cimino fistula); however, the patient was not on a hemodialysis regimen. At presentation, serum calcium was 2.32 mmol/L (reference range, 2.05–2.65), phosphate 3.7 mg/dL (2.5–4.8), creatinine 3.2 mg/dL (0.7–1.2), glomerular filtration rate (GFR) 19 mL/min (≥90), parathormone 752 pg/mL (15.0–65.0) and 25-hydroxyvitamin-D_3_ 24.6 ng/mL (20.0–100.0). ^18^F-FDG PET/CT imaging with 223 MBq ^18^F-FDG and unenhanced CT due to CKD exhibited multifocal osteolytic lesions with highly elevated glucose consumption (see Figure 1A), e.g., a large osteolytic, partly sclerotic lesion in the right ilium and the lateral surface of the right sacrum destroying the sacroiliac joint (SUV_max_ 9.8; mean 45 HU; 7.6 cm × 4.4 cm in transverse planes; see Figure 1B). Additionally, two masses with lower ^18^F-FDG uptake adjacent to the right thyroid lobe (SUV_max_ 4.6, 3.0 cm × 2.2 cm in transverse planes), as well as a right intrathyroidal mass with low ^18^F-FDG uptake (SUV_max_ 2.0, 3.0 cm × 2.3 cm in transverse planes) were found. There were no further lesions suspicious of malignancy. The presence of a parathyroid lesion with elevated ^18^F-FDG uptake is generally suspicious of parathyroid adenoma or carcinoma [1]; however, the low ^18^F-FDG uptake, especially compared with the osseous lesions, made a metastatic parathyroid carcinoma or a metastatic thyroid carcinoma rather unlikely [2]. Additionally, a ^99m^Tc-MIBI scintigraphy with 281 MBq using SPECT/CT imaging and low-dose CT comprised highly elevated tracer retention in good spatial correlation to the previously noted lesion on PET/CT (see the green arrows in Figure 1D), a characteristic finding of parathyroid adenoma. However, no MIBI-avidity of the intrathyroidal lesion was observed, a finding rather untypical for thyroid cancer [3]. A further counter-indicator for a malignant condition was the absent MIBI-avidity of the multiple lytic lesions (see the blue arrows in Figure 1D) [4]. Therefore, contrary to the suspected referral diagnosis of multiple metastatic sites, the most likely diagnosis based on ^18^F-FDG PET/CT and ^99m^Tc-MIBI SPECT/CT imaging was active parathyroid adenoma and secondary osteitis fibrosa cystica generalisata with formation of multiple brown tumors. Histopathological analysis following bone biopsy confirmed the diagnosis of benign, so-called brown tumors: H&E staining exhibited osteoclast-like giant cells (see Figure 1C), and negative G34W staining (monoclonal antibody targeting the G34W mutation in the H3F3A gene of histone H3.3. protein) excluded primary giant cell tumor of the bone; there were no signs of malignancy. Histopathological analysis following right-sided hemi-thyroidectomy confirmed the diagnosis of parathyroid adenoma; there was no evidence of parathyroid or thyroid malignancy. Postoperative serum calcium due to hungry bone syndrome was 1.67 mmol/L (reference range, 2.05–2.65), phosphate 4.5 mg/dL (2.5–4.8), creatinine 3.9 mg/dL (0.7–1.2), GFR 15 mL/min (≥90) and parathormone 104 pg/mL (15.0–65.0). Laboratory follow-up gave no signs of tertiary hyperparathyroidism. Brown tumors are a rare pitfall on ^18^F-FDG PET/CT reading due to their impressively malignant-like glucose consumption and destructive character [5,6,7]. This phenomenon has also sporadically been reported with other PET tracers [8,9]. As illustrated by the current case, molecular imaging with ^18^F-FDG PET/CT and ^99m^Tc-MIBI scintigraphy can lead to the correct differential diagnosis of osteitis fibrosa cystica generalisata.

## Data Availability

Not applicable.
